# Successful recruitment to trials: a phased approach to opening gates and building bridges

**DOI:** 10.1186/1471-2288-11-73

**Published:** 2011-05-19

**Authors:** Sue Patterson, Hilary Mairs, Rohan Borschmann

**Affiliations:** 1 Rethink, 89 Albert Embankment, London SE1 7TP, UK; 2 School of Nursing, Midwifery and Social Work, University of Manchester, UK; 3 Institute of Psychiatry, King's College, London, UK

## Abstract

**Background:**

The pragmatic randomised controlled trial is widely regarded as the gold standard method for evaluating the effectiveness of health care interventions. Successful conduct of trials and generalisation of findings depends upon efficient recruitment of representative samples, which often requires the collaboration of 'gatekeepers' who mediate access to potential participants. Effective negotiation of gatekeeping is thus vital to process and outcomes of trials and the quality of evidence. Whilst relevant literature contains discussion of the problems of recruitment and gatekeeping, little is known about how recruitment can be optimised and factors leading to successful recruitment.

**Discussion:**

As practised researchers with first-hand experience of gatekeeping, we were aware that some researchers recruit more effectively than others and curious about the ingredients of success. With the goal of developing practical guidance, we conducted a series of workshops with 19 expert researchers to investigate and map successful recruitment. Workshops were digitally recorded and transcribed. Analysis of discussion supported modelling of effective recruitment as a process involving three phases, each comprising two key tasks. Successful negotiation of set-up, alliance, and exchange require judicious deployment of interpersonal skills in an appropriately assertive manner. Researcher flexibility and credibility are vital for success, such that a foundation for rapprochement between the worlds of research and practice is established.

Our model provides a framework to support design and implementation of recruitment activities and will enable trouble shooting and support recruitment, supervision and training of effective researchers. This, in turn will support delivery of trials on time and on budget, maximising return on investment in the production of evidence.

**Summary:**

Pragmatic trials are central to development of evidence based health care but often failure to recruit the necessary sample in a timely manner means many fail or require costly extensions. Gatekeeping is implicated in this. Drawing on the knowledge of 19 expert researchers, we argue that successful researchers are resourceful and personable, judiciously deploying interpersonal skills and expertise to engage with gatekeepers and establish a shared objective. We propose that understanding recruitment as a phased process can enhance design and conduct of trials, supporting completion on time, on budget.

## Background

The pragmatic randomised controlled trial takes the lead in the theatre of evidence-based health care. Recruitment, whilst crucial to the plot, is cast in a supporting role, typically getting limelight only when performance deviates from the script. Meanwhile gatekeeping, sometimes cast as the villain of the piece, manipulates the action. Both process and outcomes of trials are affected when potential participants' capacity to be invited into a study is inhibited by others. Frequently, necessary samples are not achieved within the expected time leading to failure and/or costly extensions and loss of precision [1,2] and selection bias compromises generalisability of findings [3,4]. Whilst factors hindering recruitment have been described [4-6] and some guidance has been published [7], extant literature provides scant advice to researchers seeking to optimise recruitment practice. It is to this knowledge gap our paper is addressed. Drawing on the combined expertise of 22 experienced researchers, we re-envisage recruitment as a phased process and discuss strategies and techniques that contribute to effective recruitment. This new understanding provides a foundation for targeted improvement activities, such that return on investment in the production of evidence will be optimised and its relevance enhanced.

### Gatekeeping and recruitment

Gatekeeping occurs wherever access to someone or something is allowed or denied by a third party [8]. Because many potential research participants are regarded as 'vulnerable' in various ways, ethical approval for a study is often conditional upon researcher access to potential participants being mediated by the clinicians involved in their care. Clinicians, and their managers, are thus gatekeepers. Though powerful, these individuals are often reluctant actors in the research arena [4] and researcher access to potential participants is problematic across healthcare fields [5,6,9-11].

Committed to a vision of the National Health Service (NHS) as a research oriented organization, the United Kingdom (UK) government has invested heavily in infrastructure to support the production of evidence [12]. Clinical research networks have been established to expedite the conduct of trials and dedicated government funding has been linked to research activity [12]. If crucial studies are to be delivered "on time and on target" [12], it is vital that researchers are appropriately equipped to manage gatekeeping efficiently and effectively. However, the script is short on direction: protocols typically contain only a variant of "researchers will present the study to clinical teams and contact clinicians each week to identify potential participants" [4].

Having each experienced the trials and tribulations of gatekeeping first hand and explored the phenomenon empirically [4], we were aware that effective recruitment is considerably more complicated than such a statement might suggest. We were also aware that some sites and individual clinicians were more productive of research referrals than others and that some researchers recruited more successfully than others. Seeking to understand this and to develop guidance to support efficient recruitment, we convened three workshops (see below) to investigate successful recruitment.

#### Recruitment Solutions workshops

Nineteen researchers (see Table 1), recruited through the authors' professional networks, participated in 'Recruitment Solutions' workshops conducted during July 2010 (full details available upon request). Groups were facilitated by SP working with RB in London (groups 1 & 2) and HM in Manchester (group 3), using a solution-focused approach. Whilst allowing space to explore situations within which challenges were encountered to 'situate' solutions, participants were encouraged to reflect on exceptions (i.e. times when the problems were not present or had been overcome). These exceptions were explored with particular attention to the strategies and techniques employed. We were curious about situations in which participants had recruited successfully and which resources, both internal and external, had contributed to this success.

**Table 1:** Workshop participant demographics.

Participant	Gender	Age	Highest qualification	Job title	Years experience
1	F	37	MSc	RA	3.5
2	F	23	BSc	CSO	.5
3	F	28	MSc	RA	2
4	F	31	MSc	RA	3.5
5	F	40	BSc	CSO	1
6	F	24	BSc	CSO	2
7	F	35	MSc	RA	2
8	F	24	BSc	CSO	2
9	F	34	BSc	RA	6
10	F	28	BSc	CRC	7
11	F	33	BSc	RA	5
12	F	45	BSc	RF	15
13	F	29	MA	RA	4.5
14	F	37	PhD	RO	9
15	M	45	PhD	SL	12
16	F	50	PhD	Prof	20
17	F	25	MSc	RO	4
18	F	31	MSc	SALT	4

19	F	25	BSc	RA	4

RA = Research Assistant; CSO = Clinical Studies Officer; CRC = Clinical Research Coordinator; RF = Research Fellow; RO = Research Officer; SL = Senior Lecturer; SALT = Speech and Language Therapist; Prof = Professor

Workshops were digitally recorded and transcribed by the authors. Analysis employed a constant comparative process [13] to identify similarities and divergences in data, supported by a dialogic collaborative process involving individual and collective meaning making [4]. Whilst retaining a complete record to ensure context maintenance, data were coded in three stages. First, each author categorized data as pertaining to a challenge or solution, a researcher skill or strategy, or as extraneous (i.e. not relevant to the goals of analysis). SP and RB met to review initial coding and develop analysis by identifying themes and subthemes under each category (second stage coding). The resultant thematic framework was circulated electronically and modified following discussion with HM. SP and RB met on two further occasions, employing multiple strategies (diagramming, story boarding and scenario testing) to develop and refine the process model as presented. Analysis and interpretation continued as multiple drafts were written and commented upon by authors. Whilst the authors' experiences necessarily informed our analysis and interpretation, we adopted a dynamic, reflexive approach to data interrogation and have checked the model with various participants who have endorsed it as consistent with their experiences and the workshop discussions.

## Discussion

### Recruitment as a process

**Figure 1:**
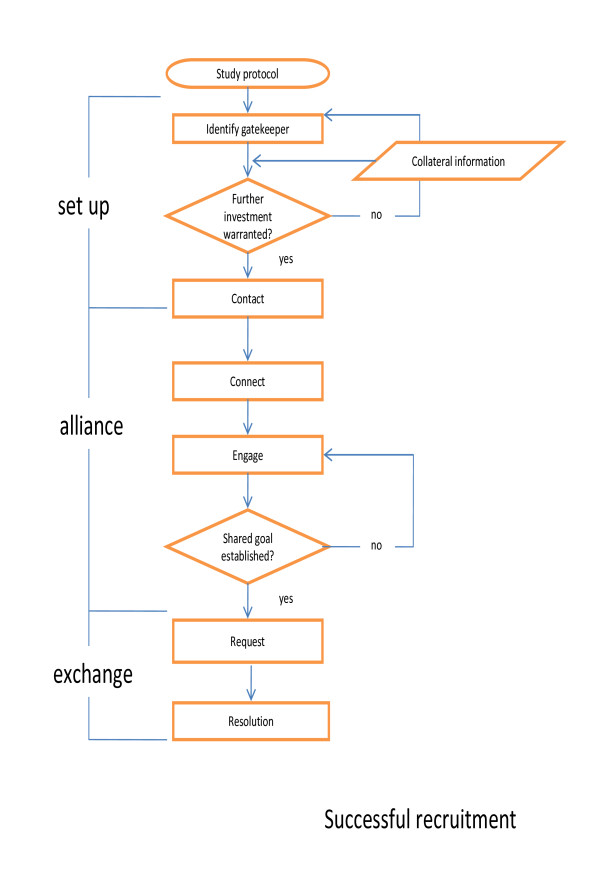
**The process of successful recruitment**.

We conceptualize successful recruitment as a process consisting of three phases - set up, alliance and exchange - each comprising two key tasks repeated with various gatekeepers, sometimes sequentially (managers, then clinicians) but often concurrently (multiple clinicians or across a number of sites). The process is developmental: satisfactory negotiation of each task is essential to effective passage and eventual resolution. This requires strategic use of resources, judicious deployment of personal and professional skills and knowledge and effective support. Importantly, it requires 'performance', vis Goffman [14] adapted to time, place, and audience. Dependent on performance, the process will be 'foreclosed' or resolved. Foreclosure occurs when tasks (particularly of the second phase) are circumvented or truncated and requests are inappropriate and/or receive indiscriminate responses. Whilst foreclosure might result in access, this is likely to be 'single entry' access and failure to engage gatekeepers intellectually in recruitment represents an opportunity lost. In contrast, resolution occurs when appropriate requests receive a considered response. Grounded as it is in meaningful dialogue, resolution contributes to sustainable relationships between researchers and gatekeepers individually and collectively. The process is described below and depicted in Figure 1.

#### 1. Set up: Identify and contact

The key tasks of set-up are to identify and make contact with the keepers of various gates which must be negotiated to gain access to the target population. Dependent on stage of recruitment, gatekeepers might be service leads, team managers, or clinicians. Because recruitment activities require investment of finite resources, cost-benefit analyses should underpin their strategic deployment. Factors to consider include the gatekeeper's capacity and authority to make the required decision and ability to facilitate necessary action. Ideally, they will be 'research savvy' and have a vested interest in supporting the study. Arguing that '*you never get anywhere with some people' *(FG2) workshop participants described using background 'intelligence' from various sources: viz, other researchers, service-based contacts, websites and the gatekeeper's colleagues to inform 'investment' decisions.

*"When you know someone is not going to refer to you... it's time to move onto to someone else. It doesn't matter what you say, they are not going to do it then... that's sort of skills as well, maximising your time and not wasting it." *(FG3)

Having identified an appropriate gatekeeper, contact must be made. In healthcare environments within which change is constant, demand for services often outstrips supply and research represents an additional burden, this requires ingenuity and persistence. Contact may most effectively be achieved with assistance of intermediaries such as administrative staff, who hold gatekeepers' contact details, diaries and facility access codes. Because *"anyone can ignore an email*" (FG1), workshop participants emphasized the importance of using multiple communication channels (understood broadly to encompass personal or professional networks), preferably simultaneously. Face-to-face contact is typically most effective, however, and can be achieved using a range of strategies dependent upon the stage of recruitment and status of the gatekeeper. Workshop participants reported achieving success as a result of 'dropping in', taking advantage of opportunistic encounters (for example, at an event) or constructing circumstances in which to approach a gatekeeper. For example, one described 'ambushing' a repeatedly unresponsive co-located clinician, having emailed her to request paracetamol for personal use. However, as described further below, caution should be exercised. Establishment of an alliance depends on maintenance of good relationships throughout these early tasks.

#### 2. Alliance: Connect and engage

Workshop participants stated that developing an effective alliance requires employment of strategies designed to win first hearts, then minds. Connecting, which begins at contact or before, and subsequently engaging with gatekeepers requires adaptability and effective deployment of communication techniques and influencing skills. As described by workshop participants, researchers need "charm with brains" (FG1) and to be "chameleon like" (FG1).

Initially, researchers must connect interpersonally with the gatekeeper. Connecting, achieved by establishing common ground - either personal or professional - begins the process of challenging the perception of 'them' (i.e. researchers perceived to have undemanding jobs) and 'us' (i.e. clinicians who do the *real *work), identified by workshop participants as hindering access and establishment of the sense of reciprocity necessary for engagement. Connection is reliant principally on the researcher's 'personality' and vitality, but cannot be divorced from the physical attractiveness and 'fit' of the researcher within the gatekeeper's environment. Superior interpersonal skills are vital. Whilst extraversion is not essential, diffidence and passivity are unlikely to ensure the researcher has the 'presence' necessary to support establishment of the common ground upon which engagement relies.

"I think just being present and making relationships with the people you work with." (FG2)

Engagement is interactive and intellectual. It is reflected in, and represents, commitment of a gatekeeper to give serious consideration to the study and opening the gate. Engagement should thus be differentiated from acquiescence, which is unlikely to support the dialogue necessary to lay the foundation for making requests in the next phase. Engagement depends fundamentally on researcher credibility, understood as a function of knowledge, personal attributes and professional conduct of the researcher and nature of the trial intervention.

Recruitment will be most effective when researchers believe in the research process and product (i.e. their study) and can demonstrate this through reasoned and critical discussion of the evidence-based approach, trial methods and the specific intervention. The researcher's knowledge must be deep enough to support translation of complex concepts (e.g. equipoise) into the language of various gatekeepers and the researcher must be confident in challenging well intentioned 'protection' [15] of potential research participants from exposure to the perceived burden of research by, for example, asserting the right of each individual to make an informed decision about participation.

"You've got to make it mean something to the clinicians. Research often doesn't mean anything to them because the benefits are years down the line." (FG1)

Relevant clinical experience might be advantageous in some (especially highly technical) circumstances, in that it enables 'shared language' (FG3) communication and facilitates expression of discipline empathy, which promotes engagement. However, humility and "knowing what you don't know" (FG1), coupled with demonstration of an interest in the concerns of the gatekeeper, are essential. Credibility is enhanced when researchers recognise and respect formal and informal politics and power structures within a site. Because failure to attend scheduled meetings and not following through with commitments impacts negatively on credibility, high level organization skills are important. At a practical level, the potential for engagement is enhanced when a researcher is co-located, or at least has regular contact, with a referring team, such that opportunistic encounters can be built upon.

Building on the sense of reciprocity established through connection, a credible researcher will be able to work with the gatekeeper to develop a shared goal, making the gatekeeper an ally in the recruitment process. Whilst ideally the shared goal would be grounded in a desire to contribute to the development of effective interventions and reflect enhanced 'sign up' to the evidence-based approach, astute researchers can also establish goals related to gatekeepers' idiosyncratic agendas.

#### 3. Exchange: Request and resolve

Once a shared goal is established, the wise researcher frames requests to fit with this and is mindful of professional culture, practice routines and competing demands on each gatekeeper's time when making such requests. Workshop participants highlighted the importance of making SMART (specific, measurable, achievable, realistic and time limited) requests [16] and, crucially, minimizing the 'bother factor' [17] by simplifying the response (and referral) procedure. When alliance is achieved a gatekeeper's responses to requests (whether positive or negative) will be informed and considered and the recruitment relationship will be secure. Resolution involves overt agreement about any further contact (e.g. for follow up); a mutually respectful, sustainable relationship will have been established. Ideally, both researcher and gatekeeper will have increased appreciation of the other's role and contribution to a shared goal - the production of evidence.

### Emotional resilience and support

"Being quite thick skinned towards it a bit, you quite often get people saying 'I don't have time for this'... they (case managers) can be very rude at times and you just can't take that personally. You've got to go back a few days later and try again." (FG3)

As the process of recruitment typically involves exposure to repeated rebuffs, successful performance is dependent on interlinked intrapersonal and external resources; effective recruitment is more likely when researchers are self-reflective, emotionally resilient and have access to responsive support. Optimal support, according to workshop participants, is both practical and emotional, provided variously by supervisors and peers within an organizational culture characterized as collaborative rather than authoritarian. Whilst acknowledging that, as individuals, they required different levels of guidance and structure from managers and that needs vary over time, participants strongly endorsed the view that line management should be grounded in respect for the challenges faced. Such respect was demonstrated by being realistic in the establishment of targets and engaging in constructive discussion of experiences (contrasted by some with providing 'solutions'). Emphasizing the importance of knowing that others shared experiences of frustration and perceived failure, colleagues' support was considered essential to management of emotional challenges linked with overt 'rejection' by potential referrers. Having empathic colleagues report similar experiences was viewed as reinforcing the notion that clinicians' actual or perceived reluctance to engage was not personal. Practical support desired by participants was most commonly related to facilitation of access to training and peer support mechanisms. Less frequently, participants reported that practical support entailed the supervisor's use of his or her 'power' or status to open gates.

### A caveat

We have, in our discussion of successful recruitment, emphasised the importance of creativity, persistence and powers of persuasion. We are acutely aware, however, that there is a fine line between being appropriately assertiveness and insufferable. Whilst some workshop participants described deliberately employing the 'nuisance factor' (FG2) and establishing removal of the irritant researcher as a shared goal, this is a risky strategy which may be more likely to lead to foreclosure than resolution.

## Summary

The vexing challenge of recruitment to trials represents a substantial impediment to the development of robust, generalisable evidence across healthcare fields. Aiming to develop guidance to promote efficient recruitment, we worked with researchers with more than 120 years experience of recruitment activity to unpack successful negotiation of gatekeeping. Drawing their experiences and insights together we have developed a process model encompassing interlinked tasks. We acknowledge that this is a fledgling model and, grounded as it is in the experiences of a self-selected sample of researchers, it is unlikely to be representative of the group as a whole, will require further development and 'testing' in various circumstances to ascertain general relevance and utility. However, we propose that, together with our insights regarding the multiplicity of skills required to negotiate recruitment tasks, the model provides a framework to support quality improvement activities. It will enable the location and troubleshooting of problem areas as suggested in NIHR guidance ([7] p.19) and contribute to thinking around recruitment, retention and training of researchers. Potentially, application of the model will provide grounds for ongoing rapprochement between the worlds of research and clinical practice. This model is a small but important step on the pathway taking recruitment "from art to science" [18].

### What is already known on this topic

• Problems recruiting trial participants are often attributed to 'gatekeeping' which occurs when access to potential participants is in the gift of others. Gatekeeping is a widely acknowledged problem across healthcare and inhibits production of evidence-based knowledge.

• If crucial trials are to be delivered on time and on target, it is vital that researchers are appropriately equipped to negotiate gatekeeping efficiently and in so doing contribute to rapprochement between the worlds of research and clinical practice.

### What this paper adds

• An understanding of successful recruitment as a phased process, negotiation of which requires timely deployment of diverse personal and professional skills

• Understanding recruitment in this way will support development and targeting of quality improvement strategies and support trouble shooting in particular circumstances.

## List of abbreviations used

FG: Focus Group; NHS: National Health Service; NIHR: National Institute for Health Research; SMART: Specific, Measurable, Achievable, Realistic, Time limited; UK: United Kingdom.

## Competing interests

All authors have completed the Unified Competing Interest form at http://www.icmje.org/coi_disclosure.pdf (available on request from the corresponding author) and declare that SP, HM and RB have no competing interests.

## Authors' contributions

SP conceptualised the paper, designed and convened the recruitment solutions workshops with RB and HM and developed the initial process model. This model refined collaboratively with RB and HM. SP wrote the paper and RB and HM contributed to drafts. All authors read and approved the final manuscript. SP is guarantor. SP and RB are both qualified as clinical psychologists and HM is an occupational therapist and cognitive behavioural therapist. All are actively engaged in research requiring negotiation of gatekeeping.

## Pre-publication history

The pre-publication history for this paper can be accessed here:

http://www.biomedcentral.com/1471-2288/11/73/prepub
